# The Evaluation of Short‐Term Outcomes and Efficacy of Robotic Complementary Gastrectomy for Remnant Gastric Cancer: A Single‐Institution Experience

**DOI:** 10.1002/wjs.70392

**Published:** 2026-05-14

**Authors:** Daisuke Fujimoto, Junpei Takashima, Hirotoshi Kobayashi

**Affiliations:** ^1^ Department of Surgery Teikyo University Hospital, Mizonokuchi Kawasaki Kanagawa Japan

## Abstract

Patient characteristics, previous gastrectomy, and clinical findings.

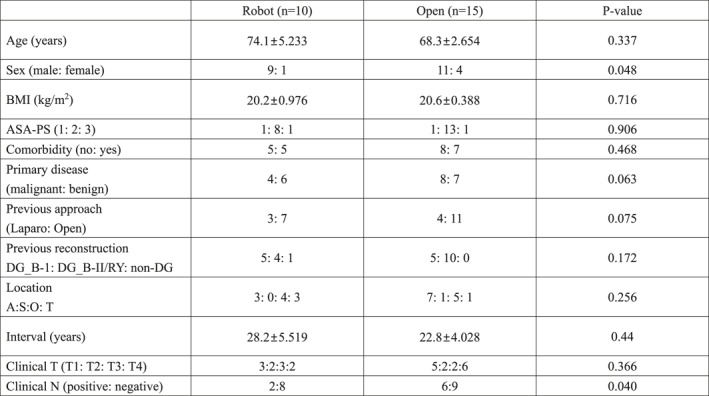

## 1 Introduction

Completion gastrectomy with radical lymphadenectomy for remnant gastric cancer (RGC) remains the standard treatment; however, the procedure is technically demanding. Anatomical distortion altered, lymphatic drainage, and dense adhesions from the prior operation contribute to increased morbidity compared with surgery for primary gastric cancer [1]. Minimally invasive surgery (MIS), including robotic gastrectomy, has become widely adopted for primary gastric cancer, offering advantages in visualization and precision [2]. Nevertheless, open completion gastrectomy (OCG) remains the predominant approach for RGC because of the technical challenges posed by adhesions and reconstruction‐related anatomical alterations. Although a few small studies have reported favorable outcomes with laparoscopic completion gastrectomy, direct comparisons between OCG and robotic completion gastrectomy (RCG) remain scarce. Therefore, this study aimed to evaluate the short‐term surgical outcomes of RCG compared with OCG in patients undergoing curative completion gastrectomy for RGC.

## 2 Methods

This retrospective study included 25 consecutive patients who underwent curative completion gastrectomy for RGC at a single institution between April 2014 and September 2024. Clinical, operative, and pathological data, which was described in accordance with the Japanese Classification of Gastric Carcinoma, 15th edition were extracted from institutional medical records. Patients with biopsy‐confirmed RGC were eligible for inclusion, whereas those with distant metastasis or requiring palliative procedures were excluded. Postoperative complications were classified according to the Clavien–Dindo system [3]. Ten patients underwent RCG, and fifteen underwent OCG. There were no specific selection criteria between the two groups. Patients with remnant gastric cancer treated from April 2019 onward underwent RCG, whereas those treated before April 2019 underwent OCG.

Robotic procedures began with open trans‐umbilical port insertion. Initial adhesiolysis was performed under direct vision prior to docking the robotic system. Our surgical approach emphasized three fundamental principles: maximizing the magnified horizontal visual field, performing ergonomic mediolateral and caudocranial manuvers, and initiating dissection in adhesion‐free areas to establish safe surgical planes. Specifically, we carefully performed adhesiolysis under a stable, magnified three‐dimensional view, taking advantage of the articulating instruments to enable precise dissection in narrow spaces. Key anatomical landmarks were identified stepwise before proceeding with lymphadenectomy, and meticulous vascular control was achieved using wristed instruments to ensure safe dissection around major vessels. We have also clarified how the robotic platform facilitates ergonomic dissection in cranial and medial directions, which is particularly advantageous in cases with dense adhesions around the remnant stomach. For example, we presented a video demonstrating the dissection technique when dense adhesions were observed between the remnant stomach and liver (Video 1). Lymphadenectomy was tailored according to the previous gastrectomy type. In all cases, perigastric lymph nodes stations (No. 2, 4sa, 5, and 6) were dissected. If the left gastric artery had been ligated at its root and lymph nodes along the superior margin of the pancreas had been dissected during a previous gastrectomy for malignant disease, lymph node dissection around the celiac and common hepatic artery was not performed. If the left gastric artery was preserved due to a benign disease, lymph node dissection around the celiac and common hepatic artery was performed. If cases where lymph node metastasis around the splenic hilum was suspected, lymph node dissection with splenectomy was performed. When the primary tumor was located at the anastomosis site in cases of B‐II reconstruction or RY reconstruction, meso‐jejunal lymph nodes were dissected. We summarized the lymph node stations dissected for each case in the supplemental table, and intracorporeal Roux‐en‐Y reconstruction was performed in all patients. All robotic procedures were conducted by experienced board‐certified surgeons. Postoperative care followed an enhanced recovery after surgery pathway, including early mobilization, standardized analgesia, and staged oral intake starting on postoperative day two.

**VIDEO 1:** Robotic‐assisted completion gastrectomy demonstrating adhesiolysis between the remnant stomach and surrounding organs. The video highlights careful dissection of dense adhesions using articulated instruments undera magnified three‐dimensional view. To view this video in the full‐text HTML version of the article, please visit https://onlinelibrary.wiley.com/doi/10.1002/wjs.70392.

## 3 Results

Baseline patient characteristics were comparable between groups, except for sex distribution (Table 1). The mean duration of follow‐up for all patients in this study was 893.7 days.

**TABLE 1:** Patient characteristics, previous gastrectomy, and clinical findings.

	Robot (*n* = 10)	Open (*n* = 15)	*p*‐value
Age (years)	74.1 ± 5.233	68.3 ± 2.654	0.337
Sex (male: female)	9: 1	11: 4	0.048
BMI (kg/m^2^)	20.2 ± 0.976	20.6 ± 0.388	0.716
ASA‐PS (1: 2: 3)	1: 8: 1	1: 13: 1	0.906
Comorbidity (no: yes)	5: 5	8: 7	0.468
Primary disease (malignant: Benign)	4: 6	8: 7	0.063
Previous approach (laparo: Open)	3: 7	4: 11	0.075
Previous reconstruction	5: 4: 1	5: 10: 0	0.172
DG_B‐1: DG_B‐II/RY: Non‐DG			
Location	3: 0: 4: 3	7: 1: 5: 1	0.256
A:S:O: T			
Interval (years)	28.2 ± 5.519	22.8 ± 4.028	0.44
Clinical T (T1: T2: T3: T4)	3:2:3:2	5:2:2:6	0.366
Clinical N (positive: Negative)	2:8	6:9	0.040

Abbreviations: A, anastomotic site; ASA‐PS, American Society of Anesthesiologists physical status; BMI, body mass index; DG, distal gastrectomy; O, other site in the stomach; S, gastric suture line; T, total remnant stomach.

No conversions to open surgery occurred in the RCG group. Operative times were similar between groups but estimated blood loss was significantly lower in the RCG group than in the OCG group (136.2 ± 40.8 mL vs. 252.1 ± 32.7 mL). Only one patient in each group required a splenectomy. Postoperative recovery outcomes favored RCG. The mean hospital stay was significantly shorter in the RCG group (13.5 ± 2.7 vs. 25.3 ± 3.1 days). Oral intake also resumed earlier in RCG patients (4.2 ± 0.2 vs. 5.7 ± 0.3 days). No mortalities occurred in either group. The overall postoperative complication rate was lower with RCG (20.0% vs. 33.3%), and notably, no severe complications (Clavien–Dindo ≥ 3a) occurred in the RCG group, whereas two such cases were reported in the OCG group. Intra‐abdominal infection occurred only in OCG patients, and pancreatic fistula was observed in one OCG patient but none in the RCG cohort. Pathological findings, including tumor depth, histology, stage, and number of retrieved lymph nodes, were comparable between groups. The mean lymph node yield and the extent of lymph node dissection did not differ significantly, underscoring the oncologic adequacy of RCG. Both the OCG and RCG groups had one case each of combined resection; both were cases where the spleen was resected concomitantly for the purpose of the dissection in splenic hilar lymph nodes (Table 2).

**TABLE 2:** Short‐term postoperative outcomes and pathological findings.

	Robot (*n* = 10)	Open (*n* = 15)	*p*‐value
Operation time (minutes)	371.7 ± 44.52	317.2 ± 17.46	0.277
Estimated blood loss (mL)	136.2 ± 40.78	252.1 ± 32.74	0.039
Hospital stays (days)	13.5 ± 2.70	25.3 ± 3.14	0.009
Time to first soft diet (days)	4.2 ± 0.2	5.7 ± 0.3	< 0.001
Postoperative complication			
Grade 1 or 2≥	2 (20%)	5 (33.3%)	0.034
Grade 3a≥	0 (0%)	2 (13.3%)	0.502
Intra‐abdominal infection	0	3	
Ileus	1	1	
Pancreatic fistula	0	1	
Wound infection	1	0	
Open conversion	0	—	
LNs retrieved (number)	13.4 ± 1.024	10.9 ± 0.993	0.098
LN dissection	10: 6: 1: 3	15: 7: 1: 5	
Perigastric: Celiac/hepatic artery: Splenic hilar/artery: Meso‐jejunal			
Histology (well: undifferentiated)	3: 7	5: 10	0.861
Pathological T (T1: T2: T3: T4)	2: 1: 3: 4	4: 4: 1: 6	0.394
Pathological N (N0: N1: N2: N3)	8: 0: 2: 0	8: 3: 0: 4	0.04
Pathological stage (IA: IB: IIA: IIB: IIIA: IIIB: IIIC)	2: 1: 3: 2: 2: 0: 0	4: 3: 1: 2: 1: 1: 3	0.396
Combined resection (+:−)	1: 9	1: 14	0.763

Abbreviation: LN, lymph node.

## 4 Discussion

In this study, we compared short‐term outcomes between RCG and OCG for RGC. The RCG group showed significantly less intraoperative blood loss, and a shorter postoperative hospital stay than the OCG group, with no cases requiring conversion to open surgery. The number of retrieved lymph nodes did not differ between groups, indicating comparable oncologic adequacy.

As the number of gastrectomies for primary gastric cancer declines, procedures for RGC have increased [1]. Although MIS, including robotic gastrectomy, is well established for primary gastric cancer, evidence supporting its use for RGC remains limited due to the rarity and technical difficulty of the disease [1, 2]. Dense adhesions to adjacent organs and difficulty distinguishing adhesions from tumor invasion make RGC surgery challenging, and inadequate adhesiolysis may result in serious complications. For these reasons, many institutions continue to prefer open surgery [4].

However, accumulating evidence suggests that robotic surgery may help overcome these difficulties [5]. The reported conversion rate during laparoscopic resection of RGC is 9.2%–14% [6, 7], whereas previous studies of RCG have reported no conversions [2, 4]. Despite the need for extensive adhesiolysis, operative time did not differ significantly between RCG and OCG, likely due to the enhanced precisions and efficiency of adhesiolysis achieved with robotic instrumentation.

The RCG group in our cohort also demonstrated lower rates of postoperative complications and intraoperative blood loss. Given losses postoperative complications and blood loss are associated with poorer long‐term outcomes [8, 9], the advantages observed with robotic surgery may translate into improved prognosis.

The major limitation of this study is the extremely small sample size inherent to this retrospective design, which may have resulted in limited statistical power and an increased risk of error. Therefore, the findings should be interpreted with caution. This study should be regarded as exploratory and hypothesis‐generating rather than definitive. To more accurately evaluate the safety and potential advantages of robotic gastrectomy for remnant gastric cancer, larger‐scale studies with longer follow‐up are warranted.

In conclusion, RCG offers favorable short‐term outcomes compared with OCG and a promising surgical option for this technically demanding disease.

## Author Contributions

**Daisuke Fujimoto:** conceptualization, formal analysis, writing – original draft, funding acquisition, project administration. **Junpei Takashima:** data curation, investigation. **Hirotoshi Kobayashi:** writing – review and editing.

## Funding

This article is supported in part by the Japan Society for the Promotion of Science KAKENHI 22K07260 (D.F.).

## Ethics Statement

All procedures performed in studies involving human participants were in accordance with the ethical standards of the institutional and/or national research committee and with the 1964 Helsinki Declaration and its later amendments or comparable ethical standards. The study protocol was approved by the Teikyo University Ethical Review Board for Medical and Health Research Involving Human Subjects (Approval No.: 21‐069).

## Consent

Informed consent was obtained from all individual participants included in the study prior to recruitment.

## Conflicts of Interest

The authors declare no conflicts of interest.

## Supporting information


**Table S1:** Dissected lymph node.

## Data Availability

Data sharing not applicable to this article as no datasets were generated or analysed during the current study.
