# Supplementary material for: The Evaluation of Short‐Term Outcomes and Efficacy of Robotic Complementary Gastrectomy for Remnant Gastric Cancer: A Single‐Institution Experience
Source: World J Surg. 2026 May 14;50(6):1685–8. doi: 10.1002/wjs.70392 (PMC13242066; doi:10.1002/wjs.70392)
Supplement: Supplementary file 2 — Table S1: Dissected lymph node. [file WJS-50-1685-s001.docx]

Supplemental table

Dissected lymph node

| Lymph node station | Robot | Open |
| --- | --- | --- |
| No.1 | 6 | 7 |
| No.2 | 9 | 15 |
| No.3 | 6 | 7 |
| No.4sa | 9 | 15 |
| No.5 | 1 | 0 |
| No.6 | 1 | 0 |
| No.7 | 6 | 7 |
| No8a | 6 | 7 |
| No.9 | 6 | 7 |
| No.11p | 6 | 7 |
| No.11d | 1 | 1 |
| No.10 | 1 | 1 |
| No.14v | 1 | 0 |
| Meso-jejunal | 3 | 5 |
